# miRNA interplay: mechanisms and consequences in cancer

**DOI:** 10.1242/dmm.047662

**Published:** 2021-04-15

**Authors:** Meredith Hill, Nham Tran

**Affiliations:** 1 School of Biomedical Engineering, Centre for Health Technologies, Faculty of Engineering and IT, The University of Technology Sydney, Sydney, NSW 2007, Australia; 2 The Sydney Head and Neck Cancer Institute, Sydney Cancer Centre, Royal Prince Alfred Hospital, Camperdown, NSW 2050, Australia

**Keywords:** RNA regulation, miRNA regulation, miRNA:miRNA interaction

## Abstract

Canonically, microRNAs (miRNAs) control mRNA expression. However, studies have shown that miRNAs are also capable of targeting non-coding RNAs, including long non-coding RNAs and miRNAs. The latter, termed a miRNA:miRNA interaction, is a form of self-regulation. In this Review, we discuss the three main modes of miRNA:miRNA regulation: direct, indirect and global interactions, and their implications in cancer biology. We also discuss the cell-type-specific nature of miRNA:miRNA interactions, current experimental approaches and bioinformatic techniques, and how these strategies are not sufficient for the identification of novel miRNA:miRNA interactions. The self-regulation of miRNAs and their impact on gene regulation has yet to be fully understood. Investigating this hidden world of miRNA self-regulation will assist in discovering novel regulatory mechanisms associated with disease pathways.

## Abstract (precis)

**Summary:** MicroRNAs are capable of self-targeting, which has implications on their biogenesis, their canonical role in gene regulation and on cancer development.

## Introduction

MicroRNAs (miRNAs) have emerged as an interesting area of basic and translational biomedical study, owing to their influence on gene expression, robust presence in bodily tissues and fluids, and their potential usefulness as disease biomarkers (Citron et al., 2017; Yoon et al., 2020). The canonical role of these small non-coding RNAs is to influence messenger RNA (mRNA) via recognition sites in the 3′untranslated region (UTR), which regulates their stability (Lee et al., 1993). miRNAs primarily affect gene expression levels via targeting mRNA. Any changes in miRNA expression may affect the extent of target regulation, and thus influence cell homeostasis (Carthew and Sontheimer, 2009; Macfarlane and Murphy, 2010). Therefore, the relative levels of miRNA, and consequently mRNA, have a major role in carcinogenesis and other diseases.

The biogenesis of miRNAs follows a series of cleavage stages in the nucleus and in the cytoplasm. The primary (pri)-miRNA transcript is cleaved in the nucleus by Microprocessor, a catalytic complex composed of Drosha and Di George critical region 8 (DGCR8) (Gregory et al., 2004; Lee et al., 2003). Recent reports have shown that the stem-looped pri-miRNA is correctly oriented for cleavage through the interaction of Drosha with the basal UG motif and alignment of the DGCR8 dimer with the apical UGU motif (Nguyen et al., 2015). Microprocessor cleavage forms precursor (pre)-miRNA, which is transported into the cytoplasm by exportin-5 (Lund et al., 2004). It is here that Dicer (also known as DICER1) cleaves pre-miRNA (Bernstein et al., 2001), and the resulting double-stranded mature miRNA is subsequently bound by Argonaute (AGO) (Song et al., 2004). The guide strand remains bound to AGO to form the miRNA-induced silencing complex (miRISC), whereas the passenger strand, denoted as miRNA*, is removed and degraded (Schwarz et al., 2003).

The main role of miRISC is to enable the RNA interference (RNAi) pathway, whereby the seed region of the miRNA, spanning nucleotides 2-8 from the 5′ end (Lewis et al., 2003), recognises Watson–Crick complementary binding sites in the 3′UTR of mRNA (Lai, 2002). Although mature miRNAs are generated in the cytoplasm, studies have shown that up 75% of known mature miRNAs are present in both the nucleus and the cytoplasm (Gagnon et al., 2014). Nuclear miRNAs, their nuclear import mechanisms and regulatory action are beyond the scope of this article and have been the topic of several other reviews (Catalanotto et al., 2016; Trabucchi and Mategot, 2019; Liu et al., 2018; Salmanidis et al., 2014).

Although the main role of miRNAs is to perform post-transcriptional gene regulation, their control of other non-coding RNAs has reshaped our understanding of RNA biology. miRNAs have been found to interact with long non-coding RNAs (lncRNAs), circular RNA (circRNA) and pseudogenes to either induce miRNA suppression or increase cellular competition for miRNA binding sites (Gebert and MacRae, 2019; Ransohoff et al., 2018; Ulitsky, 2018). In this Review, we summarise the recent studies that have demonstrated how miRNAs can in fact regulate non-coding RNAs, with a particular focus on their control of other miRNAs. The molecular process of miRNA regulation via another miRNA has been previously termed a miRNA:miRNA interaction (Hill and Tran, 2018). Here, we discuss the mechanisms behind miRNA:miRNA interactions, their role in cancer pathogenesis and the pitfalls of current investigative methods. For more information about miRNA interactions with other non-coding RNAs, we point the readers to excellent reviews on this topic (Fabbri et al., 2019; Grillone et al., 2020; Ulitsky, 2018; Anastasiadou et al., 2018).

### The discovery of miRNA:miRNA interactions

Complementary miRNA pairs in *Drosophila* were first noted in 2004, whereby Watson–Crick binding was used to identify pairing between miR-5 and miR-6, and between miR-9 and miR-79. The binding between these miRNA pairs was predicted to be stronger than that between the guide miRNA and passenger miRNA* strands (Lai et al., 2004). The authors of this study and other groups proposed that the formation of complementary miRNA pairs would increase their stability, or prevent target regulation (Lai et al., 2004; Guo et al., 2012).

The identification of these miRNA pairs was based on sequence analysis, and was not confirmed *in vitro*. Nevertheless, this study theoretically established that miRNAs could bind to other miRNAs and non-coding RNAs, and suggested how this may alter homeostatic gene regulation (Lai et al., 2004). Subsequent work, discussed below, has determined the occurrence of miRNA:miRNA interactions *in vitro* under several different mechanisms. miRNA:miRNA interactions have wide-reaching effects on cell functionality, and are believed to add another layer to miRNA and mRNA regulation.

### Direct miRNA:miRNA interactions

**Fig. 1.**
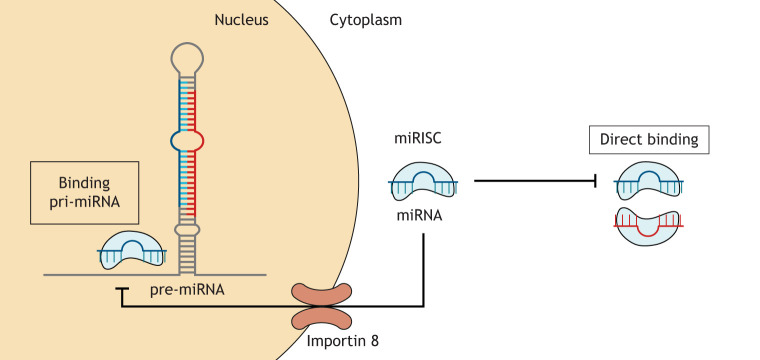
**Direct miRNA:miRNA interactions.** These occur either between two mature miRNAs in the cytoplasm or a mature and a primary miRNA hairpin in the nucleus. These nuclear interactions typically prevent the binding of Microprocessor and thus block the maturation of the primary miRNA, reducing its levels and preventing the silencing of its target mRNA. The cytoplasmic interaction between two mature miRNAs is sequence specific and brings together two miRNA-bound RNA-induced silencing complexes (miRISCs). However, the functional consequences of this interaction on miRISC activity are not fully understood.

As the term implies, direct miRNA:miRNA interactions occur when a miRNA binds another in a complementary fashion. This has been demonstrated between two mature miRNAs in the cytoplasm (Chen et al., 2011; Lai et al., 2004), or involving a mature and a pri-miRNA within the nucleus (Forrest et al., 2010; Tang et al., 2012; Wang et al., 2018a; Zisoulis et al., 2012) (Fig. 1).

### Mechanism of action

Several studies have investigated the direct binding between miRNAs as a mode of miRNA:miRNA interaction. The first of these determined that miR-424 and miR-503 both directly regulate miR-9 via recognition sites in its pri-miRNA form (Forrest et al., 2010). Although not stated directly, the targeting of pri-miR-9 implies that this particular interaction occurs within the nucleus. miR-424 and miR-503 are both classified as differentiative miRNAs, meaning that they promote cellular differentiation, whereas miR-9 is anti-differentiative. The downregulation of miR-9 by miR-424 and miR-503 thus suppresses its ability to maintain the cell in an undifferentiated state, and promotes cell lineage commitment and growth (Forrest et al., 2010).

A pivotal discovery in mice, whereby miR-709 bound the pri-miR-15a/16-1 in the nucleus to modulate its production (Tang et al., 2012), introduced the concept of a miRNA hierarchy, in which an initial group of specific miRNAs are responsible for the wide-spread post-transcriptional control of miRNAs. This induces the expression of a secondary level of miRNAs to continue the cascade of post-transcriptional regulation. This study also indicated that miRNA:miRNA interactions can influence the biogenesis pathway, and thus alter miRNA production (Tang et al., 2012).

Several important facets of miRNA:miRNA interactions were uncovered in a communication by Zisoulis et al. (2012). This study demonstrated that the mature *Caenorhabditis elegans* miRNA let-7 could bind to and regulate pri-let-7 to promote its production, forming a positive feedback loop (Zisoulis et al., 2012). Because the cleavage of primary miRNAs occurs in the nucleus, this discovery suggests that mature miRNAs can migrate to the nucleus to perform their regulatory role, sparking further investigation into nuclear miRNAs (Zisoulis et al., 2012; Liang et al., 2013). Additionally, this study also demonstrates that miRNAs can regulate their own production via the control of their immature form. As such, miRNA:miRNA interactions may have a role in auto-regulation.

Two studies focused on miRNA:miRNA interactions have demonstrated that the recognition and binding of a mature miRNA to a pri-miRNA impedes Microprocessor attachment and prevents pri-miRNA cleavage, decreasing its abundance. Analyses of murine cardiomyocytes found that the pri-miR-484 sequence contains a binding site for miR-361 within its transcript, and that this binding prevented pri-miR-484 cleavage by Drosha within the nucleus, which in turn prevented cardiomyocyte apoptosis (Wang et al., 2014). A recent report found that miR-122, which is commonly expressed in the liver, regulated miR-21 by controlling the expression of its primary transcript (Wang et al., 2018a). The miR-122 recognition site within the pri-miR-21 transcript lies within the region recognised by Drosha, and binding of miR-122 to pri-miR-21 blocks Drosha-mediated cleavage and processing, ultimately reducing the amount of mature miR-21 within the cell (Wang et al., 2018a). This mechanism has significant implications for cell growth and proliferation, as miR-21 is a known regulator of the tumour suppressor programmed cell death 4 (PDCD4) (Lu et al., 2008; Wang et al., 2018a). This was most evident in a hepatoma mouse model, in which the addition of miR-122 and mutant pri-miR-21 increased tumour growth compared to wild-type pri-miR-21. The mutation of pri-miR-21 in this case prevented miR-122 directed downregulation, and increased overall miR-21 levels to promote tumour development (Wang et al., 2018a). These examples of miRNA-binding sites within pri-miRNA sequences indicate that miRNAs acting in the nucleus may interfere with miRNA production, especially through blocking Drosha cleavage. This could indicate a wider mechanism for the regulation and coordination of miRNA expression.

Another manner of direct miRNA:miRNA interactions is through the recognition of complementary sequences within two mature miRNAs. For example, miR-107 binds to a complementary sequence within the tumour-suppressing miRNA let-7, resulting in the suppression of the mature let-7. The duplex formed by these two mature miRNAs has a series of bulges within its structure, of which the internal loop is vital for the interaction (Chen et al., 2011). However, this interaction raises questions as to how two mature miRNA may undergo binding while bound by miRISC, and the actions of the miRISC components. A study showed that amino acid residues within Argonaute 2 (AGO2) can allow miRNAs to bind to non-canonical targets and aid in miRNA cooperation (Flamand et al., 2017). Although this has not yet been tested in the context of miRNA:miRNA interactions, it may be that this mechanism is responsible for the binding of two AGO2 complexes. Additionally, several studies on miRNA:miRNA interactions have put forward the notion that they may increase the stability of miRISC. Canonical binding to a target often results in miRISC stabilisation, and non-canonical binding in its destabilisation (Park et al., 2017). Thus, direct binding of two miRNAs may aid stabilisation and the prevention of miRNA degradation.

These examples show the variability of miRNA:miRNA interactions and raise questions relating to the extent of this mode of regulation among pri-miRNA and mature miRNA. Additionally, the precise mechanisms by which nuclear miRNAs perform post-transcriptional silencing, including that of other miRNAs (Tang et al., 2012; Wang et al., 2018a, 2014; Zisoulis et al., 2012), remains to be fully understood. As multiple studies describe the binding of mature miRNAs to the pri-miRNA strands, it is possible that this binding mechanism represents a wider mode of miRNA regulation that has yet to be thoroughly explored. The mechanism behind the binding of two mature miRNAs and how this modulates the RISC components of both miRNAs has also not yet been explained.

### Impact on disease

Several direct miRNA:miRNA interactions have been implicated in disease development. The mature let-7 miRNA is controlled by miR-107. Because let-7 is a tumour suppressor, its downregulation and suppression by miR-107 leads to an increase in the abundance of its target oncogenes, contributing to downstream tumorigenesis (Chen et al., 2011). Similarly, owing to the role of miR-484 in cardiomyocyte apoptosis (Wang et al., 2012), the direct interaction between miR-361 and miR-484 has implications on cardiac diseases such as myocardial infarction (Wang et al., 2014). Additionally, the downregulation of pri-miR-9 by miR-503 and miR-484 promotes cellular lineage commitment (Forrest et al., 2010). If this interaction is disrupted, miR-9 is upregulated, leading to an undifferentiated state typical of cancer cells.

Another oncogenic miRNA, miR-21, is overexpressed in most solid malignancies. In non-cancerous liver cells, miR-21 is under miR-122-mediated inhibition, which increases the expression of the miR-21 target gene *PDCD4*, controlling cell proliferation. However, if miR-122 regulation is lost, miR-21 expression increases, leading to a decrease in PDCD4 levels and thus contributing to a cancer phenotype (Lu et al., 2008; Wang et al., 2018a). miR-21 upregulation affects cell proliferation and size, and allows for the continued growth and survival of cancer cells. Therefore, the miRNA:miRNA interaction between miR-122 and pri-miR-21 is vital in controlling cellular homeostasis, the cell cycle and the prevention of oncogenic changes.

As many of the miRNA:miRNA interactions discussed involve the transportation of a mature miRNA to the nucleus to regulate a pri-miRNA, it is important to determine whether miRNA transport is altered in cancerous cells. A disruption in nuclear import of miRNAs would prevent pri-miRNA targeting, and may alter the expression of their target miRNAs and mRNAs, thus adding to the cascade of oncogenic alterations. An example of this has already been demonstrated, whereby the knockdown of importin 8 prevented miR-709 transport into the nucleus, subsequently increasing the levels of miR-15a/16-1 (Wei et al., 2014). Further studies are recommended to determine the nuclear and cytoplasmic distribution of miRNAs in cancer cells compared to normal physiological levels to assess whether there is an impact on miRNA and mRNA expression.

### Indirect miRNA:miRNA interactions

**Fig. 2.**
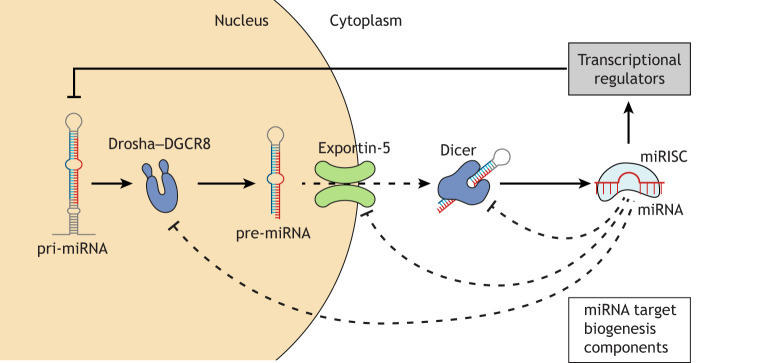
**Indirect miRNA:miRNA interactions.** These interactions occur through miRNA-directed suppression of the miRNA biogenesis pathway components or transcriptional regulators. The suppression of the biogenesis components has consequences on the production of specific miRNAs, rather than the expected negative effect on overall miRNA production. Targeted transcriptional regulators may include transcription factors, DNA methyltransferases and repressors.

Although the several studies discussed have shown that miRNAs are capable of directly regulating miRNAs at different stages of their biogenesis, miRNA:miRNA interactions can also occur through indirect means (Fig. 2).

### Role of transcription factors

One such miRNA:miRNA interaction pathway is the control of transcription and its impact on miRNA production. In this model, miRNAs target the 3′UTRs of mRNAs encoding transcriptional regulators, such as transcription and methylation factors, to induce changes in their expression. In this way, a miRNA can modulate the expression of another miRNA by controlling its transcription or regulatory pathways as part of a gene regulatory network (Song et al., 2015). Consequently, this miRNA:miRNA interaction is caused by secondary transcriptional control, rather than a direct interaction.

The first example of such regulatory network was demonstrated in murine adult cardiac muscle cells, whereby miR-208a modulated the transcription of miR-208b and miR-499 (van Rooij et al., 2009). Here, the miRNAs are encoded within the introns of various myosin genes. miR-208a, encoded within a fast myosin gene, is capable of negatively regulating the repressors responsible for silencing the expression of slow myosin gene transcripts containing miR-499 and miR-208b. An increase in miR-208a reduces the availability of slow myosin gene repressors, and thus an upregulation in miR-499 and miR-208b. In the heart, miR-208b upregulation requires the additional presence of stress signals such as calcium or hypothyroidism, but the activation of miR-499 does not require outside stimulus. An increase in these miRNAs induces the expression of slow muscle genes via the targeting of repressors. Activation of the slow muscle genes amplifies the signal for the expression of the genes containing miR-499 and miR-208b. This ultimately forms a positive feedback loop that allows for accurate modulation of miRNA levels with respect to alterations in the physiological environment and thus for the regulation of muscle contractility (van Rooij et al., 2009). This was the first study to introduce the concept of miRNA modulation via the miRNA-mediated control of transcription factors and repressors (van Rooij et al., 2009; Zhang and Zeng, 2010).

An auto-regulatory loop has been discovered involving miR-20a and the transcription factors of the E2 factor (E2F) family, which are essential cell cycle and apoptosis regulators. In this feedback mechanism, the miR-17-92 family, containing miR-20a, regulates the expression of the E2F genes (Sylvestre et al., 2007). Simultaneously, the E2F members E2F1, E2F2 and E2F3 activate the expression of miR-20a by binding to its promoter. In this way, an increase in miR-20a levels suppresses the production of the E2F transcription factors, subsequently decreasing miR-20a transcription. The authors proposed that the primary role of this mechanism is to modulate the expression of the E2F genes to prevent apoptosis (Sylvestre et al., 2007). However, this feedback loop also highlights indirect miRNA auto-regulation mediated by transcription factors.

A recent study in lung cancer cells found that the tumour suppressor miR-660-5p controls the expression of miR-486-5p via mouse double minute 2 (MDM2) and p53 (also known as TP53) (Borzi et al., 2017). In this model, miR-660 silences its direct target *MDM2*, which consequently results in an increase in p53 (Borzi et al., 2017). Because p53 is a transcription factor involved in miRNA biogenesis, and is a potent tumour suppressor, its activation upon MDM2 silencing initiates the transcription of miR-486-5p, miR-29 and the miR-34 family (Borzi et al., 2017). Therefore, this network demonstrates the wider impact of miRNA:miRNA modulation via their control of transcriptional regulation.

In addition to the modulation of transcription factors, miRNAs may affect the production of other miRNAs by inducing changes in epigenetic markers. A study in tongue squamous cell carcinoma tissues demonstrated that miR-29b downregulates the DNA methyltransferase gene *DMNT3B*, which in turn alters the methylation pattern of the miR-195 promoter. This induces an increase in miR-195 production, generating a positive regulatory system in which upregulation of miR-29b increases the levels of miR-195. As both miRNAs are tumour suppressors that are downregulated in cancer, this mechanism may offer a therapeutic window for tongue squamous cell carcinomas (Jia et al., 2016). These examples show how indirect control of miRNAs via transcription factors, promoters and epigenetics has wider implications on miRNA expression, and the capacity to influence several cellular pathways, including those in cancer development (Ali Syeda et al., 2020).

### Role of miRNA biogenesis components

miRNAs can regulate the expression of miRNA biogenesis pathway components, which been shown to affect the production of several miRNAs and which may affect the overall abundance of miRNAs in a cellular system. A study in epithelial ovarian cancer showed that miR-98-5p can regulate the expression of miR-152 by targeting the mRNA transcript of Dicer, forming an indirect miRNA:miRNA interaction (Wang et al., 2018b). This study demonstrated that miR-152 levels change in response to both miR-98 overexpression and Dicer knockdown. However, owing to the involvement of Dicer in this pathway, it would be expected that the expression levels of most miRNAs in this system would change (Song and Rossi, 2017), and that this mode of regulation would not be limited to miR-152.

Another study has also investigated an indirect miRNA:miRNA interaction involving the biogenesis pathway member AGO2 (Leonov et al., 2015). Within human dermal lymphatic endothelial cells, miR-132 suppressed AGO2 when activated by phorbol myristate acetate (PMA). Conversely, inhibition of miR-132 resulted in an increase in AGO2. PMA activation of miR-132 also resulted in a decrease in miR-221 and an increase in miR-146a, and subsequent inhibition of miR-132 elevated miR-221 and miR-146a. These miRNAs demonstrated a decreased mature-to-pre-miRNA ratio in response to decreased AGO2, meaning that their mature strands were less abundant (Leonov et al., 2015). However, the study also highlighted that other regulatory mechanisms may also contribute to the observed changes in miR-221 and miR-146a (Leonov et al., 2015). The interactions between these miRNAs have consequences on inflammation and angiogenesis, as the pro-angiogenic miR-132 promotes a decrease in the anti-angiogenic miR-221 and an increase in the inflammatory miR-146a (Leonov et al., 2015).

These findings highlight that, although the downregulation of the miRNA biogenesis pathway components by miRNAs themselves may result in a global decrease in miRNA abundance, researchers more commonly observe that this mechanism only affects select miRNAs. For several members of the biogenesis pathway, such as Drosha, miRNA target sites have yet to be experimentally validated (Chou et al., 2018; Kishore et al., 2011). It is suggested that if Drosha was negatively regulated by miRNAs, this would have a miRNAome-wide impact owing to its key role in miRNA production. It is also apparent across the literature that there is a lack of understanding of the overall effect that alterations in miRNAs and their production has on cellular interactions and functioning.

### Impact in cancer

Several miRNA:miRNA interactions are integrated into pathways that are critical to cancer progression. Such interactions include that between miR-205 and miR-184, which mediates the levels of the lipid phosphatase SH2-containing phosphoinositide 5′-phosphate 2 (SHIP2; also known as INPPL1) (Yu et al., 2008). Both these miRNAs have overlapping binding sites within the 3′UTR of *SHIP2*, whereby miR-184 mediates miR-205-driven suppression of SHIP2 by blocking access to the binding site without inducing regulation. However, an increase in miR-205 and a decrease in miR-184, which thus also decreases SHIP2, are observed in cancer, particularly in corneal squamous cell carcinoma (Yu et al., 2008). This has implications on cellular proliferation, growth and apoptosis owing to the involvement of SHIP2 in the AKT pathway, implying that this miRNA:miRNA interaction is a major contributor to the cancerous phenotype.

The previously described miRNA:miRNA interaction involving miR-660-5p, MDM2 and miR-486-5p was proposed as a potential target for lung cancer therapy via the stabilisation of the tumour suppressor p53 (Borzi et al., 2017). p53 is, among its other functions, involved in the PI3K-AKT pathway, and is commonly dysregulated in cancer. As such, the disruption of this pathway results in p53 instability, which has downstream effects on cancer development. Borzi et al. (2017) proposed that induction of miR-660-5p could be a potential therapeutic, as its suppression of MDM2 would effectively stabilise p53, reducing tumour growth.

Similarly, the indirect interaction between miR-98 and miR-152 through the regulation of Dicer discussed above (Wang et al., 2018b) has implications on chemotherapy resistance in epithelial ovarian cancer. In this cancer type, high levels of miR-98 were observed in conjunction with low miR-152 levels, which results in the upregulation of the DNA repair gene *RAD51*, promoting chemotherapy resistance (Wang et al., 2018b). Mouse *in vivo* models showed that tumours treated with miR-152 and the chemotherapeutic agent cisplatin were significantly smaller and showed decreased cell proliferation compared to those that were treated with miR-152 or cisplatin alone (Wang et al., 2018b). This study demonstrates that miRNA:miRNA interactions also contribute to the morphology and the therapy-resistant characteristics of cancer cells.

The oncogenic miRNA miR-21 has been found to be involved in several miRNA:miRNA interactions; for example, perpetuating tumorigenic changes through its indirect regulation of miR-145 expression in colon cancer (Yu et al., 2015). An increase in miR-21 initiates K-Ras signalling, activating the transcription factor Ras-responsive element binding protein (RREBP; also known as RREB1), which in turn inhibits the transcription of miR-145 (Yu et al., 2015). Therefore, the increase in miR-21 observed in cancer results in the decreased expression of miR-145, amplifying oncogenic changes. Additionally, miR-21 levels are influenced by the targeting of its primary strand by miR-122 to prevent oncogenic changes in liver cells (Wang et al., 2018a).

### Global miRNA:miRNA interactions

**Fig. 3.**
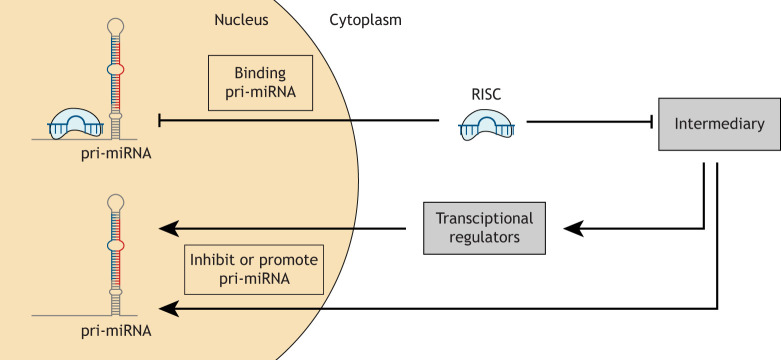
**Global miRNA:miRNA interactions are due to the culmination of the reactions within the cell that control miRNA expression.** These consider all direct and indirect changes in miRNA and mRNA expression in response to a perturbation in miRNA expression. Full comprehension of the complexity of miRNA:miRNA interactions in a cellular system involves the integration of several mechanisms, and the consideration of resultant secondary changes in miRNA and mRNA.

We have discussed the idea that one miRNA could modulate the expression of several others, or an entire miRNA family (Borzi et al., 2017). However, little research has focused on the impact of a miRNA on the global miRNA expression in a cellular system (Fig. 3).

Higher-order miRNA:miRNA interactions were addressed in murine cardiac cells in a seminal study by Matkovich et al. (2013), in which the downstream miRNA and mRNA changes were measured in response to miR-499 and miR-378. Transgenic overexpression of miR-499 upregulated 11 miRNAs and downregulated six miRNAs, whereas miR-378 upregulated 18 miRNAs and downregulated 31 miRNAs. The results suggest that both miR-499 and miR-378 influence the transcription of other cardiac miRNAs, although not directly, as the stability and the guide-to-passenger strand ratio of the target miRNAs were not affected (Matkovich et al., 2013). Of the affected miRNAs, 13 were encoded within genes that were targeted directly by miR-499 or miR-378, and were thus co-regulated in the transgenic models, explaining the mechanism behind a fraction of the regulated miRNAs. It is suggested that the remaining changes in miRNAs were as the result of miR-499 and miR-378 target deregulation. It was noted that miR-378 suppresses the MAF and RORA transcription factors, resulting in a decrease in miR-99. As a consequence, 31 miR-99 targets are deregulated indirectly by miR-378 (Matkovich et al., 2013). The authors also found that, in the miR-499 model, 76 downregulated mRNAs (7.8%) were targets of miR-499 and 298 (31%) were targets of the upregulated miRNAs. It was suggested that the remaining 595 (75%) downregulated mRNAs were the result of secondary miRNA changes. This was an instrumental study for the field, as it established that alterations in miRNA levels have a global impact on the miRNA environment, resulting in secondary mRNA and miRNA changes. Thus, this study broadens our understanding of the mechanisms that drive indirect miRNA:miRNA interactions.

As we have discussed above, miRNAs have been investigated in terms of their indirect regulation of target transcripts via their influence on miRNA expression. Shahab et al. (2012) overexpressed miR-7 in ovarian cancer cells and analysed the changes in both miRNA and mRNA expression levels. They identified secondary regulated genes within the cellular milieu (Shahab et al., 2012). However, the question remains as to how the introduction of a miRNA can influence downstream miRNA levels in both an indirect and direct manner. Several theories have arisen on the wider impact of individual miRNA changes on the miRNAome, which include a change in promoter activity downstream from the miRNA genomic coding region, the inclusion of miRNA sequences within dysregulated genes, or the influence of altered transcription factor activity (Shahab et al., 2012).

Recent studies have observed that a miRNA may adjust the expression of another miRNA to amplify the regulatory effect on a common target. The miR-130/301 family expression levels are increased in pulmonary hypertension, resulting in a decrease in miR-204, miR-322 and miR-503 via peroxisome proliferator-activated receptor gamma (PPARG) and signal transducer and activator of transcription 3 (STAT3) (Bertero et al., 2014). Under hypoxic conditions, an elevation in the miR-130/301 family results in a targeted decrease in PPARG. Within pulmonary arterial smooth muscle cells, this induces an elevation in STAT3 and a consequent decrease in miR-204 expression, resulting in an increase in cell proliferation. Additionally, in pulmonary arterial endothelial cells, PPARG represses apelin as well as miR-424 and miR-503, also increasing cell proliferation. Together, these two pathways elevate endothelial and smooth muscle cell proliferation, leading to pulmonary hypertension phenotypes. The changes observed in the miRNAs and the consequences they have on cell proliferation indicate that a number of miRNAs may act cooperatively to drive molecular changes to a greater effect than the actions of individual miRNAs (Bertero et al., 2014).

The concept of miRNA synergism implies the presence of a ‘master regulator’ miRNA, a miRNA that influences the majority of miRNAs within the cell system. Thus, any changes to the expression of the master regulator miRNA would also alter the miRNAs within its synergistic network. Similarly, miRNAs that target transcriptional regulators may alter the transcriptional activity of miRNAs that are similar in function to aid in a coordinated response (Ooi et al., 2017).

However, very few studies investigate this miRNA:miRNA interaction phenomenon, particularly in cancer cells. Given its large overall impact on the miRNA and mRNA environment, changes to master regulator and synergistic miRNAs could have dire consequences for the cell, and may affect the many cellular pathways that are altered in cancer. Therefore, it is important that global miRNA:miRNA interactions are investigated in cancer cell systems.

### miRNA:miRNA interactions in disease

Many of the examples discussed in the sections above have been observed and tested in the context of cancer. However, several questions still remain as to the nature of miRNA:miRNA interactions, the mechanisms behind their dysregulation, and the understanding of their impact in the context of chemotherapeutic resistance.

### Exclusivity to cell type

Given that miRNA and mRNA expression are tied to the cell type, it can be assumed that miRNA:miRNA regulatory networks also convey this specificity. Nuclear miRNA distribution is also dictated by cell type, and hence extends to the range of nuclear miRNA:miRNA interactions (Salmanidis et al., 2014). Current prediction algorithms do not take this distinction into account (Rock et al., 2019). As a consequence, information from miRNA:target interaction databases may not convey the cell type investigated, which may lead to inaccurate conclusions when mining the data. These inaccuracies also affect the genes and miRNAs used to map miRNA:miRNA networks. The network from one cell type cannot be used to infer that of another. At present, the cell specificity of target information and miRNA prediction is an ongoing area of research, and investigators mining the existing databases should consider cell specificity as a key factor.

### Mechanism behind dysregulation

At present, there is no one theory or mechanism for the dysregulation of miRNAs in cancer. It is possible, given the complexity of physiological systems, that multiple mechanisms are at play, including those involving the miRNA biogenesis components, transcription factor regulation and mutations within miRNA strands.

Aberrations in the miRNA biogenesis pathway components affect miRNA expression. A recent report showed that mutations within the RNase IIIb domain of Dicer depleted 5p-stranded miRNAs, which affected the ratio of 3p-to-5p mature miRNA (Vedanayagam et al., 2019). Alterations to the 3p-to-5p ratio change the spectrum of targeted genes, such as in endometrial cancer, where patients with Dicer mutations had derepressed genes that contained sites enriched for the let-7, miR-17, miR-15/16, miR-29 and miR-101 families. Although rare, mutations in Dicer were also observed in several other cancers, including bladder, kidney and uterine carcinomas, and may only provide a selective advantage in particular tissues (Vedanayagam et al., 2019). This study invokes the question of how the miRNA:miRNA network is altered in response to changes in strand selection, as this affects the expression of target genes and downstream transcription factors and miRNAs.

It is also important to consider whether inhibition of miRNA transport into the nucleus influences the degree to which pri-miRNAs or gene promoters are targeted by miRNAs. In the case of exportin-5 loss-of-function mutations, pre-miRNAs are incapable of transportation into the cytoplasm, resulting in a decrease in mature miRNA levels (Kim et al., 2016). Consequently, it is hypothesised that a reduction in mature miRNA may affect miRNA:miRNA interactions in both cellular compartments, and consequently contribute to the cancer phenotype (Hata and Kashima, 2016).

On a genome-wide scale, the loss or gain of super-enhancers, which are genomic loci that contain multiple enhancer elements and that collectively bind multiple transcription factors, has extensive repercussions on miRNA and gene expression (Suzuki et al., 2017). Under normal physiological conditions, super-enhancers control the transcription of genes and miRNAs that dictate cell type. If altered, this drives a loss of cell specificity, typical of carcinogenesis (Matsuyama and Suzuki, 2019). A decrease in the miRNAs that determine cell type results in an increase of miRNAs that were previously expressed at lower levels. Consequently, this altered miRNAome controls a different set of genes, adding further to potential oncogenic changes (Li et al., 2018). Typically, the loss of super-enhancer regions results in an increase in tumour suppressor miRNAs, whereas a gain in super-enhancers enriches oncogenic miRNAs (Suzuki et al., 2017). It is imperative, then, that future investigations into miRNA:miRNA interactions are approached at a systems-wide level to gain a greater understanding of the changes that may occur to miRNA expression and their targets (Matsuyama and Suzuki, 2019).

Another factor that drives changes in miRNA expression are single-nucleotide polymorphisms (SNPs) within their seed region (Lewis et al., 2003). The seed sequence is essential for binding to a target mRNA, or target recognition sequence. Changes to either of these domains may result in a loss of target regulation. Additionally, different miRNA isoforms (IsomiRs) may alter the seed region via the addition of nucleotides from the 5′ or 3′ end of the miRNA. IsomiRs have implications in gene targeting, and have roles in disease development (Bofill-De Ros et al., 2020). The presence of SNPs or IsomiRs that change the seed region have the potential to alter both mRNA and miRNA expression, which would have cascading effects on the cellular milieu (Króliczewski et al., 2018). The extent to which the miRNA seed sequence participates in miRNA:miRNA interactions is currently unknown. However, it is suggested that alterations in this region may disrupt miRNA–mRNA–miRNA networks.

### Aid in developing therapeutics

Understanding the interplay between miRNAs and their impact on gene expression is integral to the exploration of potential cancer therapeutics and their off-target effects (Lapa et al., 2019). Several reports on miRNA:miRNA interactions have studied these networks in the context of their response to chemotherapeutic agents, such as that to the Erb-B2 receptor tyrosine kinase 2 (ERBB2) inhibitor Trastuzumab in breast cancer (Cilek et al., 2017), cisplatin resistance in ovarian cancer (Wang et al., 2018b) or experimental anti-miRNA agents, like miR-34 (Ooi et al., 2017). Further investigation of miRNA:miRNA interactions in cancer and other diseases will benefit both our mechanistic understanding of these diseases and aid in the identification of viable therapeutic targets.

### Role of bioinformatics

Bioinformatics approaches have had a major role in investigating the impact of miRNA:miRNA interactions. Several studies have combined databases pertaining to gene, miRNA and lncRNA interactions in a network (Liu and Ye, 2019; Ulitsky, 2018; Zhao et al., 2008). This generates a greater understanding of the coding and non-coding genetic players that may drive disease.

For example, as a given miRNA alters the expression of mRNA, this may in turn alter the expression of downstream miRNAs. The formation of a miRNA–mRNA–miRNA network may be used to identify a master regulator miRNA that controls the expression of most miRNAs within the network (Hu et al., 2020; Ooi et al., 2017), as exemplified by miR-1 having been identified using bioinformatics as a potential master regulator miRNA in prostate cancer (Alshalalfa, 2012). Clearly, bioinformatics is an important technical approach in understanding the interplay between different RNA species and in the identification of master regulator miRNAs and the miRNA hierarchy (Bertero et al., 2014).

The interactions between miRNAs have also been determined by identifying those with overlapping subpathways (Wu et al., 2013). Here, the authors used computational tools to show that miR-21 has more connections to downregulated miRNAs than to upregulated ones. Again, this may affect the direct pathway that miR-21 and the other miRNAs in the analysis are involved in, but also indirectly influence other subpathways via these miRNAs (Wu et al., 2013).

However, one issue to continually consider is the lack of information pertaining to the cell specificity of miRNA and mRNA expression and interaction. This encompasses the miRNAs present and active in the nucleus and cytoplasm, as well as cell-specific IsomiRs (Salmanidis et al., 2014). Current algorithms that predict miRNA binding, such as miRanda (Betel et al., 2010), do not account for tissue or cell type of origin, which may skew bioinformatic and experimental analyses (Rock et al., 2019). Cell-specific variations in miRNA sequences also add extra complications to the identification of miRNA targets and miRNA:miRNA interactions (Glogovitis et al., 2021). Additionally, findings that are exclusively based on bioinformatic analyses should be confirmed with *in vitro* experimentation (Liu and Ye, 2019).

Many studies investigating the wider impact of miRNAs on controlling cell processes use miRNA sequencing (miRNAseq) or miRNA array methods. Current array methods only identify annotated miRNAs of high confidence, whereas miRNAseq has been utilised to identify novel miRNAs and IsomiRs, especially those that are cell-type specific. Therefore, paired with RNAseq, miRNAseq is the preferred method for determining changes in miRNAs and the levels of their respective targets, which can subsequently be used for network analysis.

As discussed, several direct miRNA:miRNA interactions involve a mature miRNA recognising a binding region within a pri-miRNA strand (Tang et al., 2012; Wang et al., 2018a; Zisoulis et al., 2012). Although the precursor sequence of a miRNA is known and well annotated, the sequence of each miRNA's primary sequence is relatively unknown. Many studies have attempted to define a library of pri-miRNA sequences, but this has proven difficult owing to the highly transient nature of pri-miRNAs, with several studies using a Drosha-dependent sequencing protocol (Kim et al., 2017). Currently, up to 20% of all known miRNAs have not been shown to have a pri-miRNA motif or a fully identified pri-miRNA sequence (Auyeung et al., 2013). Researchers also targeted the pri-miRNA strand by designing primers 100 bp upstream and downstream from the precursor strand (Conrad et al., 2020) to define the pri-miRNA sequence itself (Wang et al., 2018a). However, this approach limits the potential for the identification of regulatory elements, including miRNA binding sites, which may be located beyond the region specified by the chosen primers.

Bioinformatic analysis of miRNAseq and RNAseq libraries is invaluable to the discovery of miRNA:miRNA interactions and their cellular implications. However, researchers should carefully consider the limitations and shortcomings of current methods, and validate findings with experimentation in living systems.

## Conclusions

The range of miRNA:miRNA interactions discussed in this Review extends to the context of specific cancer environments. Although many cancer types exhibit similar traits, the expression of miRNAs, miRNA:miRNA regulatory pathways, and the extent of target suppression are specific to the cell type of origin (Matsuyama and Suzuki, 2019; Shao et al., 2019). Therefore, caution must be taken in both investigating miRNA:miRNA interactions and applying the findings broadly, as specific regulatory pathways mediated by miRNA-to-miRNA associations may not be the same in other cell types.

The current strategies of investigating miRNA:miRNA interactions usually involve the transfection of a miRNA mimic or antisense inhibitor. Any conclusions based on these approaches need to be made with caution, as the introduction of an exogenous miRNA inherently alters endogenous miRNA and mRNA expression (Khan et al., 2009). Comparisons should be made to a scramble miRNA control to identify biologically relevant changes. An alternative may be to regulate the miRNA at the primary or precursor transcript stage to avoid saturating AGO2. Another possibility is to use aptamers or longer antisense strands to sequester endogenous miRNAs. Future experimentation should consider how to determine miRNA:miRNA interactions and their effects on cell functioning without drastically altering the delicate balance of endogenous miRNA and mRNA.

At present, not many miRNA studies have considered the wider impact of miRNA on overall miRNA expression. The realm of miRNA:miRNA interactions often focuses on a particular pair of miRNAs, or a small subset, rather than on the changes that occur in the miRNA milieu. miRNA and mRNA alterations that result from miRNA:miRNA interactions have been demonstrated to affect cell growth and metastasis (Borzi et al., 2017; Wang et al., 2018a). By taking into account one or a few miRNA:miRNA interactions, we are ignoring the systems-level impact that is inherent to miRNA-mediated regulation.

In summary, miRNA:miRNA interactions, especially those encompassing the miRNA and mRNA milieu, require a re-evaluation, and this added regulatory pathway may underpin or drive a better understanding of disease mechanisms. Interestingly, the presence of miRNAs in the nucleus and their potential for targeting pri-miRNA indicates that they may have a wider role in gene regulation than the canonical model of targeting the 3′UTR of mRNA. We can no longer hold the simple notion that a single miRNA may regulate several targets. Instead, this must be extended to incorporate the idea that miRNAs may regulate each other. As miRNAs are potent regulators and have been shown to drive oncogenic pathways, the impact of miRNA:miRNA interactions could be profound. Moving forward, the community must be mindful of the effects of miRNA networks in studies pertaining to the role of miRNAs in cancer and beyond, and their application in therapeutics.
